# Atypical presentation of Lemierre’s syndrome: case report and literature review

**DOI:** 10.1186/s12879-019-4538-6

**Published:** 2019-10-21

**Authors:** Marie-Eva Laurencet, Sarah Rosset--Zufferey, Jacques Schrenzel

**Affiliations:** 1 Service of General and Internal Medicine, Department of Medicine, Geneva University Hospitals, Geneva, Switzerland; 2 Service of Infectious Diseases, Department of Medicine, Geneva University Hospitals and the University of Geneva, 4 Rue Gabrielle-Perret-Gentil, 1211 Geneva 14, Switzerland; 3 Bacteriology Laboratory, Department of Diagnostics, Geneva University Hospitals, Geneva, Switzerland

**Keywords:** Atypical Lemierre syndrome, *Fusobacterium*, Septic thrombophlebitis

## Abstract

**Background:**

The classic Lemierre’s syndrome refers to a septic thrombosis of the internal jugular vein, usually caused by a *Fusobacterium necrophorum* infection starting in the oral cavity, and typically complicated by pulmonary emboli. However, unusual forms of the disorder have been rarely reported.

**Case presentation:**

We describe an unusual case of a previously healthy 58-year-old male with Lemierre’s syndrome, manifesting with lumbar pain and fever. A thrombosis of the iliac veins and abscesses in the right iliac and the left psoas muscles was diagnosed by a computed tomography scan, together with a right lung pneumonia complicated by pleural effusion and an L4-L5 spondylodiscitis. Blood culture and pus drainage were positive for *Fusobacterium nucleatum* and an atypical Lemierre’s syndrome was suspected. The patient was treated with anticoagulant therapy for 12 weeks and intravenous antibiotic therapy for 6 weeks with a good evolution and resolution of the thrombosis.

**Conclusions:**

This case illustrates the thrombogenic and thromboembolic tendency of *Fusobacterium nucleatum* and its potential invasiveness, regardless of the site of primary infection. The concept of an atypical Lemierre’s syndrome is redefined here to take into consideration non-cervical sites.

## Background

The definition of Lemierre’s syndrome remains controversial. It was initially described by André Lemierre in 1936 as an “anaerobic postanginal septicemia”, most often due to *Fusobacterium necrophorum* and responsible for thrombosis of the superior internal jugular vein paralleled with embolic abscesses. However, the syndrome can also be considered during anaerobic septicemia originating from diverse sources of infection, such as the upper respiratory tract, the gastrointestinal system or the genitourinary tract [1]. Of note, it is necessary to differentiate septic cases with thrombophlebitis from (un) complicated bacteremia due to *Fusobacterium*, the so-called “necrobacillosis” (Table 1) [1, 2].

**Table 1:** Examples of atypical Lemierre’s syndrome

Age, gender	Location of the infection	Organism	Atypical presentation	Reference, year
A) Complicated bacteremia due to *Fusobacterium (necrobacilloses)*				
42 yr, female	Vertebral osteomyelitis	*F. nucleatum*	Complicated bacteremia without thrombosis or metastatic abscess	Ramos et al., 2013 [2]
B) Conditions mimicking a Lemierre syndrome				
4 yr, male child	Multiple peri-tonsillar abscesses with brain, orbits and lung emboli	*Staphylococcus aureus*	Organism mimicking Lemierre’s syndrome	Aouad et al., 2010 [3]
59 yr, male	Right internal jugular vein thrombosis and abscesses	*Streptococcus anginosus*	Organism mimicking Lemierre’s syndrome	Osman et al., 2017 [4]
C) Atypical Lemierre’s syndrome				
26 yr, male	Lower limb trauma complicated by extensive inferior vena cava and femoral vein thrombosis, lung abscesses	*F. necrophorum*	Lower limb origin, site of thrombosis (inferior vena cava)	Razonable et al., 2003 [5]
47 yr, female	Left ovarian vein thrombosis (with intrauterine device)	*F. necrophorum*	No obvious origin of infection, site of thrombosis (ovarian vein)	Huynh-Moynot et al., 2011 [6]
32 yr, male	Prostatic abscess (on urinary catheter) with iliac vein thrombosis, pulmonary abscess and pleural fistula	*F. necrophorum*	Prostatic origin, site of thrombosis (iliac vein)	Bonny et al., 2019
26 yr, male	Inferior vena cava and common femoral vein thrombosis and multiple abscesses in the lungs	*F. necrophorum*	Site of thrombosis (femoral vein thrombosis) After a trauma-associated abscess of the limb	Razonable et al., 2003 [5]
58 yr, male	Thromboses of the two iliac veins, abscesses in muscles, pleural effusion complicating a pneumonia and L4-L5 spondylodiscitis	*F. nucleatum*	Site of thrombosis (iliac veins), site of abscesses (iliac and psoas muscle), spondylodiscitis and organism	This report

Gram-negative anaerobic bacilli involved in Lemierre‘s syndrome are mostly *F. necrophorum* or *F. nucleatum,* but *F. gonodiaformans, Bacteroides fragilis* and *B. melaninogenicus* have been also reported [4]. The risk factors that trigger the invasive process are not clearly known, but they appear to depend upon the location of the initial infection. Patients with a post-anginal septicemia are generally young and healthy, although some authors have postulated a previous viral infection or a damage of the oral mucosa related to tobacco consumption. The gastrointestinal and genitourinary sources of infection seem to develop in elderly patients with a higher risk of an underlying malignant disease. Indeed, any digestive mucosal injury, e.g. due to cancer or diverticulitis, promotes the risk of bacterial translocation. Finally, preterm delivery and chorioamnionitis constitute a third group of specific obstetrical conditions favoring the development of anaerobic septicemia [5, 7].

## Case presentation

A previously healthy 58-year-old male was transferred from another hospital to our department due to sepsis of undetermined origin with an unfavorable evolution under broad-spectrum antibiotic therapy (imipenem-cilastatin and clarithromycin). The patient complained of pain in the lumbar region and fever throughout the preceding two weeks. He also described asthenia, anorexia and a recent weight loss of 3 kg. The medical history was unremarkable. He was a non-smoker and reported no drug abuse, recent travel or contact with animals.

Upon admission to our hospital, the patient presented a high fever of 39.3 °C with hemodynamic stability. The physical examination showed no heart murmur or signs of cardiac congestion, but the presence of bilateral painless swollen legs was observed. Pulmonary auscultation showed respiratory crackles on the right side. The neurological, abdominal, cutaneous and osteoarticular examinations were unremarkable.

Laboratory examinations revealed the following results: hemoglobin 116 g/l (normal range: 140–180 g/l); leukocyte count, 33.3*10^9^ cells/l (4–11*10^9^ cells/l) without left-band shift; C-reactive protein, 114.6 mg/l (0–10 mg/l); creatinine, 56 μmol/l (62–106 μmol/l), aspartate transaminase, 174 U/l (12–50 U/l); alanine aminotransferase, 209 U/l (14–50 U/l); alkaline phosphatase, 406 U/l (25–102 U/l); gamma-glutamyl transpeptidase, 517 U/l (9–40 U); total bilirubin, 76 μmol/l (7–25 μmol/l); conjugated bilirubin, 66 μmol/l (0.5–9.5 μmol/l); International Normalized Ratio, 1.38. Serology assays for hepatitis B virus, hepatitis C virus and human immunodeficiency virus were negative. Urinary sediment was unremarkable.

**Fig. 1:**
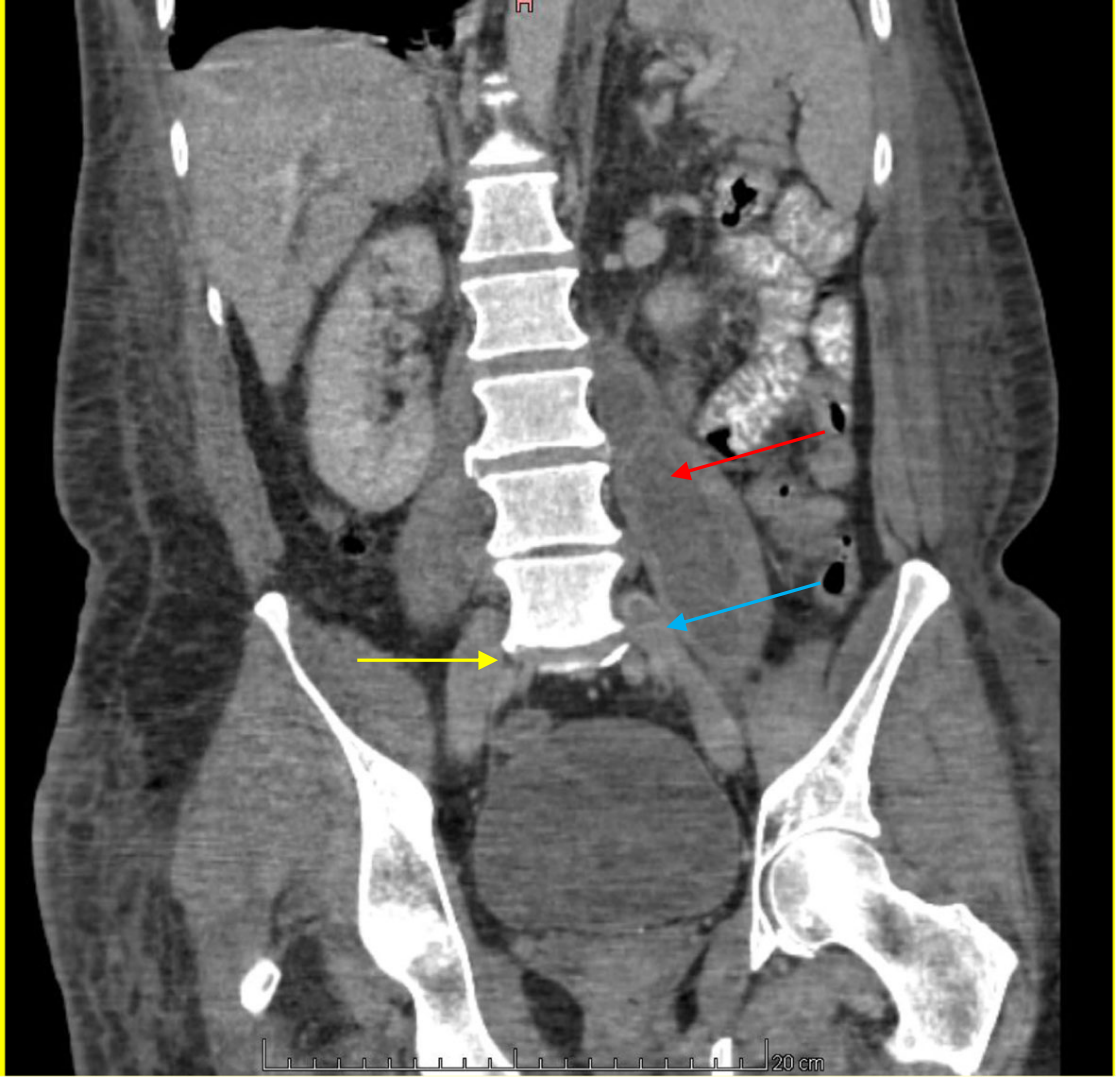
Frontal view of the CT scan. The yellow arrow shows L4-L5 spondylodiscitis, the red arrow shows the left iliac abscess and the blue arrow shows the left iliac thrombosis

As there was a high suspicion of spondylodiscitis, blood cultures were ordered and a thoraco-abdominal computed tomography (CT) scan was performed. The scan revealed thromboses of the two iliac veins, abscesses in the right iliac muscle (2.3 × 2.0 cm) and the left psoas muscle (6.0 × 4.8 cm), a right pleural effusion complicating a pneumonia, as well as a L4-L5 spondylodiscitis (Fig. 1). Lumbar magnetic resonance imaging confirmed an L4-L5 spondylodiscitis and the presence of abscesses in the muscles. One blood culture (taken initially as an outpatient before antibiotic administration) and the culture of the pus drained from the right psoas muscle grew *F. nucleatum*. The right pleural effusion was also drained and confirmed the presence of an empyema without bacterial growth (under antimicrobial therapy). Antibiotic therapy was then switched to amoxicillin-clavulanate in combination initially with clindamycin as *F. nucleatum* is pan-susceptible to these antimicrobials.

When considering the presence of *F. nucleatum* bacteremia with a complicated pneumonia, a spondylodiscitis and multiple muscle abscesses, we suspected a case of Lemierre’s syndrome and attemted to identify the source of the infection. An orthopantomogram, a transoesophageal echocardiography as well as a cerebral CT scan were unremarkable. The duplex sonography confirmed bilateral venous iliac thromboses. As the patient presented lower gastrointestinal bleeding under anticoagulation (acenocoumarol), a colonoscopy was performed, but revealed no sign of malignancy or mucosal lesions. An inferior vena cava filter was put in place and withdrawn at one month and anticoagulation was continued for a total duration of 3 months. Antibiotic treatment was given intravenously for 6 weeks. At 3 months, duplex ultrasound showed complete resolution of the thromboses and clinical follow-up was normal. Spinal magnetic resonance imaging at 9 months showed sequelae of the L4-L5 spondylodiscitis.

We searched PubMed using the following terms “atypical + Lemierre syndrome” and then selected the atypical cases as illustrations. The search was not exhaustive.

## Discussion and conclusions

Whereas a typical Lemierre’s syndrome consists of a septic cervical thrombophlebitis, usually complicated by septic emboli, atypical presentations have been reported in the abdomen, either in the context of genitourinary infections [8] or related to other intra-abdominal [9] or lower limb infections [5] (Table 1). Based on the absence of septic thrombophlebitis, these cases should be formally considered as different from bacteremia due to *Fusobacterium* [6, 10]. In our case, the presentation and evolution of Lemierre’s syndrome were atypical, as well as the identification of the less frequent *F. nucleatum* [8]. The source of infection was likely of colonic origin due to the lower gastrointestinal bleeding and the occurrence of two iliac thromboses.

The second observation in our case was the thromboembolic behavior of *Fusobacterium* spp., probably due to the production of endotoxins, which promote platelet aggregation [11]. The localization of the thromboses in the iliac veins was also atypical, but it was most likely related to the proximity of the site of infection, as reported in other cases [5, 7]. The use of anticoagulant therapy is controversial in Lemierre’s syndrome. Some authors propose to introduce anticoagulation in the case of antibiotic failure or when thrombosis extends further [1]. However, due to the severity of the infection in our patient and the swollen legs, we decided to treat the septic iliac thrombophlebitis with a 3-month course of anticoagulants.

In summary, treatment of Lemierre’s syndrome typically consists of surgical drainage of the abscess, if present, and intravenous antibiotic therapy for 4–6 weeks, although the overall treatment duration is not well established. Antibiotic therapy with anaerobic coverage must be rapidly introduced. Mortality is difficult to estimate, but can be high (up to 25%) and depends on the timing of antibiotic initiation [4, 7] . As penicillin-resistant strains have been reported, empiric therapy should consist of clindamycin or metronidazole or the use of a combination of beta-lactams with beta-lactamase inhibitors [3].

## Data Availability

Not applicable.

## Abbreviation

CT: Computed tomography
